# Mean arterial blood pressure: potential predictive tool for preeclampsia in a cohort of healthy nulliparous pregnant women

**DOI:** 10.1186/s12884-019-2580-4

**Published:** 2019-12-03

**Authors:** Jussara Mayrink, Renato T. Souza, Francisco E. Feitosa, Edilberto A. Rocha Filho, Débora F. Leite, Janete Vettorazzi, Iracema M. Calderon, Maria L. Costa, Louise Kenny, Philip Baker, Jose G. Cecatti, for the Preterm SAMBA study group, Mary A. Parpinelli, Karayna G. Fernandes, José P. Guida, Danielly Santana, Rafael B. F. Galvao, Bianca F. Cassettari, Lucia Pfitscher, Daisy Lucena de Feitosa, Elias de Melo Ferreira Júnior, Danilo Anacleto, Vilma Zotareli, Marcia Alice Silva

**Affiliations:** 1 Department of Obstetrics and Gynecology, University of Campinas (UNICAMP) School of Medical Sciences, Rua Alexander Fleming, 101, Campinas, SP 13083-891 Brazil; 2 MEAC, Maternity Hospital of the Federal University of Ceará, Fortaleza, CE Brazil; 3 Department of Maternal and Child Health, Maternity Hospital, Federal University of Pernambuco, Recife, PE Brazil; 4 Department of Obstetrics and Gynecology, Maternity Hospital, Federal University of RS, Porto Alegre, RS Brazil; 5 Department of Obstetrics and Gynecology, Botucatu Medical School, Unesp, Botucatu, SP Brazil; 6 College of Life Sciences, University of Leicester, Leicester, UK; 7 Faculty of Health and Life Sciences, Department of Women’s and Children’s Health, Institute of Translational Medicine, University of Liverpool, Liverpool, UK

**Keywords:** Preeclampsia, Blood pressure, Hypertension, Prenatal screening, Second trimester, Third trimester

## Abstract

**Background:**

Prediction of preeclampsia is a challenge to overcome. The vast majority of prospective studies in large general obstetric populations have failed in the purpose of obtain a useful and effective model of prediction, sometimes based on complex tools unavaible in areas where the incidence of preeclampsia is the highest. The goal of this study was to assess mean arterial blood pressure (MAP) levels at 19–21, 27–29 and 37–39 weeks of gestation and performance of screening by MAP for the prediction of preeclampsia in a Brazilian cohort of healthy nulliparous pregnant women.

**Methods:**

This was a cohort approach to a secondary analysis of the Preterm SAMBA study. Mean arterial blood pressure was evaluated at three different time periods during pregnancy. Groups with early-onset preeclampsia, late-onset preeclampsia and normotension were compared. Increments in mean arterial blood pressure between 20 and 27 weeks and 20 and 37 weeks of gestation were also calculated for the three groups studied. The accuracy of mean arterial blood pressure in the prediction of preeclampsia was determined by ROC curves.

**Results:**

Of the 1373 participants enrolled, complete data were available for 1165. The incidence of preeclampsia was 7.5%. Women with early-onset preeclampsia had higher mean arterial blood pressure levels at 20 weeks of gestation, compared to the normotensive group. Women with late-onset preeclampsia had higher mean arterial blood pressure levels at 37 weeks of gestation, than the normotensive groups and higher increases in this marker between 20 and 37 weeks of gestation. Based on ROC curves, the predictive performance of mean arterial blood pressure was higher at 37 weeks of gestation, with an area under the curve of 0.771.

**Conclusion:**

As an isolated marker for the prediction of preeclampsia, the performance of mean arterial blood pressure was low in a healthy nulliparous pregnant women group. Considering that early-onset preeclampsia cases had higher mean arterial blood pressure levels at 20 weeks of gestation, future studies with larger cohorts that combine multiple markers are needed for the development of a preeclampsia prediction model.

## Background

The prediction of preeclampsia is challenging. It is a complex syndrome, with multiple phenotypes, each with its own particularities of pathophysiology and clinical manifestations [1]. Effective prediction of the condition would represent an important strategy against adverse outcomes of maternal and perinatal health.

The majority of prospective studies in large general obstetric populations have demonstrated a modest capacity to predict preeclampsia by clinical risk factors, and approximately only a third of these cases are identified [2]. A prediction model using a combination of biomarkers and uterine artery Doppler has improved the diagnostic rate in early-onset cases, where disease manifestation occurs before 34 weeks of gestation. However, late-onset cases of preeclampsia (with manifestation occurring at or after 34 weeks) are the majority of cases in most clinical settings. The application of such an expensive technological screening model in low and middle-income countries is unfeasible, exactly the locations where there is a higher prevalence of preeclampsia [2–4].

Blood pressure measurement is part of routine surveillance during antenatal care. High blood pressure may be the first sign of a hypertensive disorder and is a diagnostic tool. Oscillations in BP measurements in a pregnant woman may reflect a trend for hypertensive disorder, and is a predictive test [5]. Since the sixties, several second-trimester studies have been reported on the use of blood pressure measurement for preeclampsia screening. There have been contradictory results concerning detection rates, which range from 8 to 93%. These different results were due to distinct diagnostic concepts of preeclampsia, diverse methods of population screening and also cutoff values used to define a positive screening test [6]. Futhermore, very few studies that specifically enrol nulliparous pregnant women have been carried out. There is actually a lack of information on this group, which is considered to be at high risk for preeclampsia [7]. The strongest known risk factor, which is a personal history of preeclampsia cannot be applied to this particular group of nulliparous women [3].

Thus, to assess the accuracy of mean arterial blood pressure (MAP) in a group of nulliparous pregnant women, this study showed the distribution of MAP levels at 19–21, 27–29 and 37–39 weeks of gestation and evaluated the accuracy of MAP at these three different time periods (basically second and third semesters) as a predictor of preeclampsia.

## Methods

This was a secondary analysis of the Preterm SAMBA study, a multicenter cohort study performed in 5 different centers in Brazil. From July 2015 to March 2018, 1200 healthy nulliparous pregnant women were enroled and received follow-up during prenatal care, including only singleton pregnancies, without any fetal malformations or previous chronic maternal disease [8]. Ethical approval for the study was obtained from relevant institutional review boards and competent authorities of each center where the study was conducted. More detailed information on the study design and methods used in this study have already been previously published [9].

### Participants and procedures

Criteria for participant enrollment were nulliparous women with singleton pregnancies between 19 and 21 weeks of gestation. Exclusion criteria included: previous history of chronic hypertension, use of medication, fetal malformations, diabetes mellitus, nephropathy, autoimmune diseases (systemic erythematous lupus or antiphospholipid syndrome), sickle cell disease, uterine malformations, previous cervical surgery, previous cerclage, history of 3 or more abortions, HIV infection, chronic use of corticosteroids or aspirin or calcium above 1 g/day or fish oil above 2.7 g/day or vitamin C above 1000 mg/day or vitamin E above 400 UI/day or heparin. This criteria is in accordance with another cohort study published previously [9].

At least three routine hospital visits were scheduled. Systolic and diastolic blood pressure of the women were measured, according to standard clinical procedure on the 3 occasions: at 19–21 weeks, 27–29 weeks and 37–39 weeks of gestation, using a manual sphyngomanometer, calibrated according standard procedures and using the same model in all participating centres. In these 3 occasions information about proteinuria was obtained based on a regular urinalysis performed in the routine prenatal care (in the first trimester and in the third trimester).

During the first visit, maternal characteristics and medical history were recorded. In addition, blood and hair samples were collected and stored appropriately in a biobank for subsequent analysis by metabolomics technology. Gestational age was estimated from the date of the last menstrual period and confirmed by an early ultrasonography performed before 20 weeks. For each scheduled visit, blood pressure was measured 3 times. Women were allowed to rest for 15 min before the first blood pressure measurement was performed. Between blood pressure measurements, the investigator waited for at least 2 min. During the examination, participants remained in a sitting position, with their right arm supported at the level of their heart. An adult blood pressure cuff was used, selecting the proper size for each participant. Pressure reading at phase V of Korotkoff sounds corresponded to diastolic pressure. Mean arterial blood pressure was obtained by the equation (2DBP + SBP)/3. The mean blood pressure at each gestational age for the three measurements was obtained by the average of three mean blood pressure measurements [BP_m_ = (BP1_m_ + BP2_m_ + BP3_m_)/3]. We also calculated the difference in mean blood pressure with measurements at 19–21 weeks and 27–29 weeks and measurements at 19–21 weeks and 37–39 weeks. Calculation was made in two steps: first, the difference was determined for each woman; and second, the mean difference was calculated.

### Outcome

Preeclampsia was the main outcome of this analysis. It was defined as the onset of hypertension (systolic blood pressure of 140 mmHg or more and/or diastolic blood pressure of 90 mmHg or more) after 20 weeks of gestation, measured on at least two different occasions, in conjunction with proteinuria (≥300 mg/day or at least 1 g/L [1+] on dipstick testing or spot urine protein/creatinine ≥30 mg/mmol [0.3 mg/mg]) or any signs of organ dysfunction [10]. Systemic complications were defined as: hematological complications (thrombocytopenia, disseminated intravascular coagulation or hemolysis); hepatic dysfunction (elevated transaminases); neurological dysfunction (examples include eclampsia, altered mental status, blindness, stroke or more commonly hyperreflexia when accompanied by clonus, severe headache when accompanied by hyperreflexia, persistent visual scotomata); renal dysfunction (creatinine ≥1.2 mg/dL) [10].

After delivery, each woman was classified as having a normal pregnancy (control group) or preeclampsia (case group). Cases were categorized into early-onset preeclampsia (women who developed preeclampsia before 34 weeks of gestation) and late-onset preeclampsia (women who developed preeclampsia after 34 + 1 weeks of gestation) [11] .

### Statistical analysis

Initially, the three groups were compared regarding sociodemographic characteristics of women using a Chi-square design-based test. Mean arterial blood pressure was then compared among the three groups (early-onset preeclampsia, late-onset preeclampsia and normotensive) using Student’s t-test. The mean difference in MBP measured at 27 and 37 weeks was estimated and compared to values at 20 weeks. Finally, we checked to see whether mean arterial blood pressure had any predictive power at three time periods (20, 27 or 37 weeks of gestation) by comparing the area under the receiver-operating characteristic curves (AUROC). Analyses were performed using SPSS and Stata software.

## Results

**Fig. 1:**
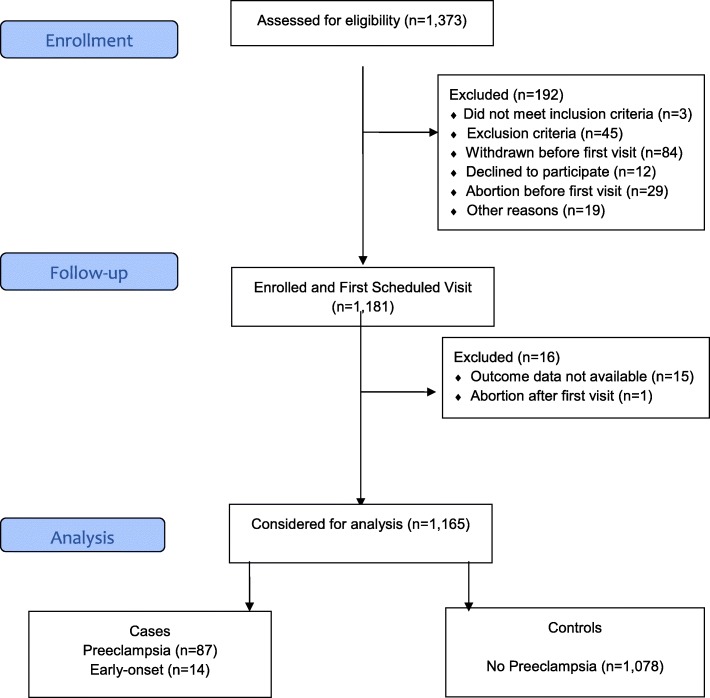
Flowchart of women participating in the study

**Fig. 2:**
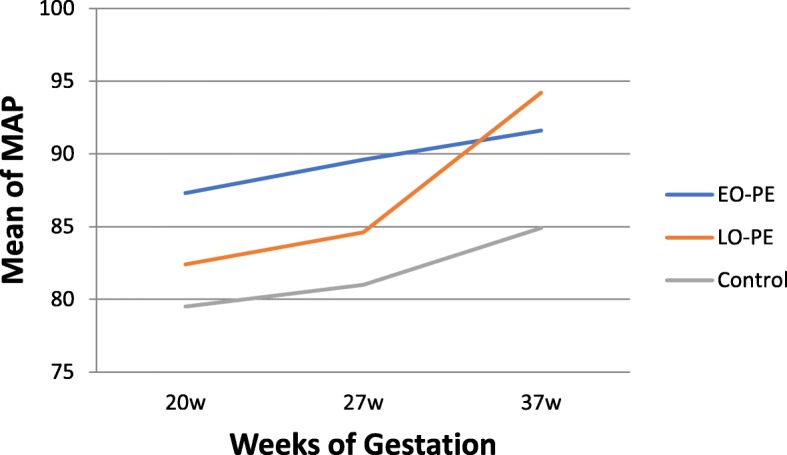
Patterns of Mean Arterial Pressure (MAP) throughout gestation in the three groups: early-onset (EO), late-onset (LO) preeclampsia (PE) groups and control group

Of the 1373 participants recruited for Preterm SAMBA study, follow-up of 1165 women was provided (Fig. 1). In our cohort, the incidence of preeclampsia was 7.5% (87 cases) of whom 14 (16.1%) had early-onset preeclampsia (data not shown). The sociodemographic characteristics of women who developed preeclampsia and were analyzed according to subtypes and controls are shown in Table 1. There were no differences between preeclampsia and control groups. Throughout the gestation period, we observed that MAP showed an increasing trend in the three participating groups (Fig. 2). MAP in the early-onset preeclampsia group showed the highest value at 20 weeks of gestation, compared to the control group (*p* value = 0.02) (Table 2). Specifically, the increment was higher in the late-onset preeclampsia group compared to the control group, in both stages of gestation: from 20 to 27 weeks and from 20 to 37 weeks of gestation, with a *p* value of 0.012 and 0.003, respectively (Table 2).

**Table 1:** Some sociodemographic characteristics of women included according to PE status

Characteristics	Early-onset PE n (%)	Late-onset PE n (%)	No PE n (%)	*p* value*
Maternal age				0.605
< 20 years	1 (7.1)	19 (26.0)	271 (25.1)	
20–34 years	12 (85.8)	48 (65.8)	736 (68.3)	
> 34 years	1 (7.1)	6 (8.2)	71 (6.6)	
Ethnicity				0.146
White	7 (50.0)	20 (27.4)	435 (40.4)	
Others	7 (50.0)	53 (72.6)	643 (59.6)	
Marital status ^a^				0.975
With partner	10 (71.4)	53 (72.6)	777 (72.4)	
Without partner	4 (28.6)	20 (27.4)	296 (27.6)	
Schooling (years)				0.717
Up to 12	8 (57.1)	50 (68.5)	733 (68.0)	
≥ 12	6 (42.9)	23 (31.5)	345 (32.0)	
Annual family income				0.691
Up to 3000 US$	3 (21.4)	21 (28.8)	280 (26.0)	
3000–6000 US$	4 (28.6)	27 (37.0)	350 (32.5)	
> 6000 US$	7 (50.0)	25 (34.2)	448 (41.5)	
Source of prenatal care				0.473
Entirely public	14 (100.0)	67 (91.8)	927 (86.0)	
Private/mixed	0 (−)	6 (8.2)	151 (14.0)	
Total	14	73	1078	

*p-value from Chi-square design-based

^a^ missing information for 5 cases

**Table 2:** Mean arterial blood pressure in the three time periods during pregnancy comparing preeclampsia groups and control

BP (mmHg)	Early-onset PE	Late-onset PE	No PE	*p*-value#	*p*-value @	*p*-value&
MBP at 20^h^ weeks ^a^ (95%CI)	87.3 (79.4–95.3)	82.4 (80.4–84.3)	79.5 (76.8–82.2)	0.191	**0.024**	0.068
MBP at 27 weeks ^b^ (95%CI)	89.6 (78.0–101.1)	84.6 (82.3–86.9)	81.0 (78.2–83.9)	0.300	0.060	0.072
MBP_27w_-MBP_20w_ y(z)	2.8 (3.2%)	4.2 (5.0%)	1.4 (1.7%)	0.100	**0.040**	**0.012**
MBP at 37 weeks ^c^ (95%CI)	91.6 (81.0–102.1)	94.2 (92.4–96.0)	84.9 (82.3–87.6)	0.581	0.218	**< 0.001**
MBP_37w_-MBP_20w_ y(z)	1.2 (1.3%)	13.3 (16.1%)	5.2 (6.5%)	**0.031**	0.229	**0.003**
Total	14	73	1078			

Missing information for: a: 1 case; b: 229 cases; c: 393 cases (125 already delivered)

y: mean difference atz: increment in percentage

# Early-onset PE x Late-onset PE; @ Early-onset PE x No PE; & Late-onset PE x No PE

*p*-values in bold mean they are statistically significant (<0.05)

**Fig. 3:**
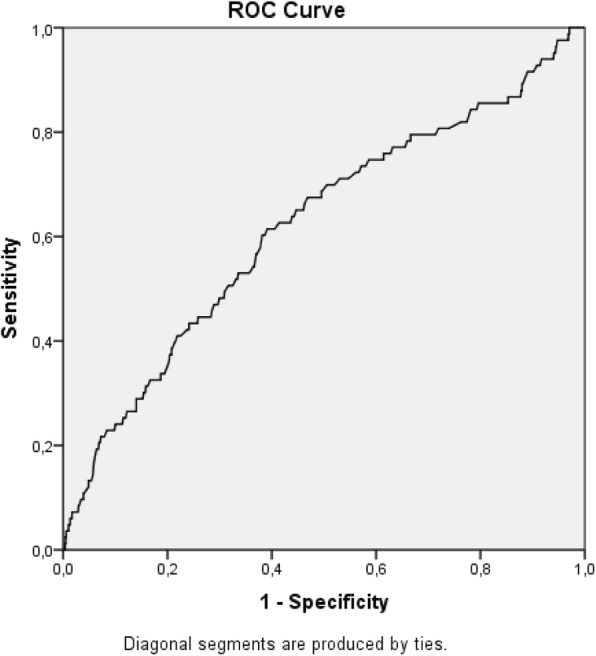
ROC curve for mean blood pressure at 20 weeks as a predictor of preeclampsia (AUC = 0.619)

**Fig. 4:**
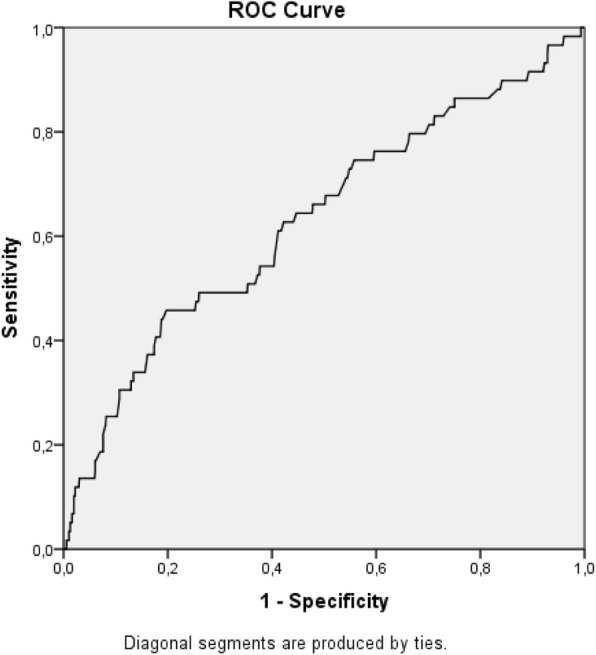
ROC curve for mean blood pressure at 27 weeks as a predictor of preeclampsia (AUC = 0.630)

**Fig. 5:**
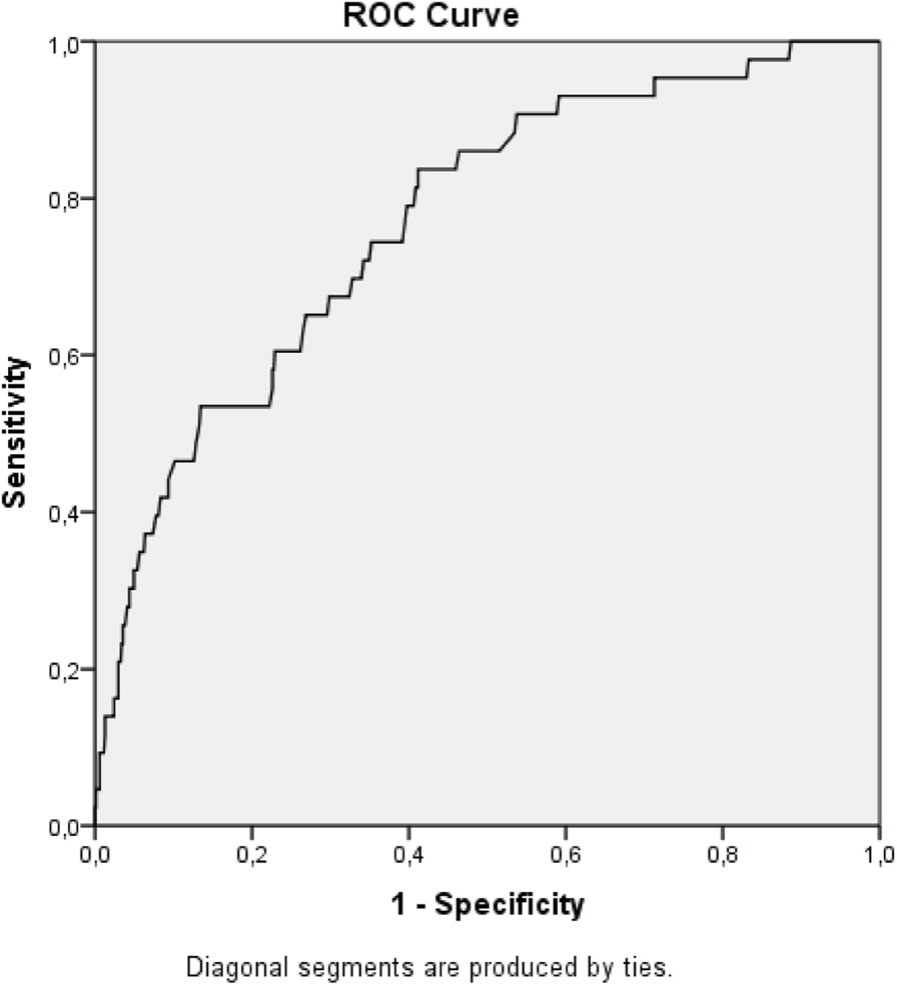
ROC curve for mean blood pressure at 37 weeks as a predictor of preeclampsia (AUC = 0.771)

When compared to the early-onset preeclampsia group, there was no difference in increment. The predictive power of mean arterial blood pressure was assessed through ROC curves, and this marker showed the highest accuracy at 37 weeks of gestation with an area under the curve of 0.771 (Table 3 and Figs. 3, 4, 5). In our cohort, there were 2 cases of eclampsia and 6 cases of HELLP syndrome, characterized by hemolysis, low platelet count and elevated hepatic transaminases (data not shown).

**Table 3:** Prediction of preeclampsia using mean blood pressure at different gestational ages among low-risk nullipara women

Gestational Age	Area Under the Curve	+/−
20 weeks	0.619	83/1048
27 weeks	0.630	59/857
37 weeks	0.771	43/707

## Discussion

Mean arterial blood pressure (MAP) has remained the target of scientific research in quest for the prediction of preeclampsia over time. It is a feasible tool and part of antenatal surveillance. Few studies have shown blood pressure patterns among low- risk nulliparous pregnant women [12–15]. Our cohort study of nulliparous low-risk pregnant women showed patterns of mean arterial blood pressure measured during the second half of pregnancy. The observation of arterial blood pressure distribution throughout pregnancy is an essential component of antenatal care strategy. This marker can be obtained from medical records usually gathered from prenatal cards that are easily available in health care services around the world.

In uncomplicated pregnancies, arterial blood pressure pattern usually consists of a steady decrease in blood pressure during the first half of pregnancy, then an increase until the time of delivery [16]. In contrast, in women with hypertensive disorder (gestational hypertension or preeclampsia) blood pressure is generally stable during the first half of pregnancy, increasing until delivery. In 2001, a study analyzing more than 2000 series of blood pressure systematically sampled by ambulatory monitoring showed that while diastolic blood pressure increases 7% between the middle of gestation and delivery in the normotensive group, this increment is around 12 and 15% in the hypertensive group [17]. Similarly, in our study there was an increment of 5.2% in mean arterial blood pressure in the normotensive group, compared to 13.3% in the late-onset preeclampsia group, with a significant difference between both (*p* = 0.003). In our study, the increment observed among late-onset preeclampsia cases was superior to the value seen among the early-onset preeclampsia group. We hypothesized that it was possible that the latter group had already started antihypertensive medication at 27 and 37 weeks of gestation. The lack of information about the exact time when antihypertensive medication was initiated was a weakness of our study.

Although we demonstrated that women with early-onset preeclampsia had a higher MAP at 20 weeks of gestation than the remaining participants, MAP performance was modest as a predictor, with an Area Under the Curve of only 0.619. This is not in accordance with a systematic review from 2008, which demonstrated that mean arterial blood pressure measured during the first or second trimester of gestation in a general low-risk pregnant population was a better predictor of preeclampsia, with an Area Under the Curve of 0.76. For the high-risk pregnant women group, this review indicated that diastolic blood pressure is the best predictor of preeclampsia, when measured between 13 and 20 weeks of gestation [18]. The very few cases of preeclampsia may possibly explain the modest accuracy found in our cohort, specially the very few cases of early-onset preeclampsia. In our analysis, the highest accuracy was achieved at 37 weeks of gestation, with an AUC of 0.771. Despite this number, prophylatic measures are totally unfeasible at this gestational age, considering the pathophysiology of preeclampsia. Regardless of the time period when preeclampsia is clinically established, it may be triggered in earlier stages of gestation [19, 20].

Furthermore, some studies have demonstrated a higher predictive power when MAP is measured during the first trimester of pregnancy [6, 15, 21], particulary when combined with other maternal factors. Participants were enrolled during the second trimester of gestation. Therefore, such data is not available, representing a weakness of our study. However, a cohort of more than 70,000 pregnant women found a similar detection rate of preeclampsia, with MAP measurement at 11–13 weeks or 19–24 weeks of gestation [22]. It is already known that the introduction of low-dose aspirin before 16 weeks of gestation in a group at high-risk for preeclampsia can reduce the incidence of EO-PE by almost 62% [23, 24]. This reinforces the importance of a proposed pregnancy care model in early pregnancy to identify possible life-threatening maternal and fetal health conditions [25]. Nevertheless, this should not reduce the significance attributed to a “second-look” prediction in the middle of gestation - in the second trimester - considering that it is still possible to redefine pregnancy management at this time period, including the frequency of visits, addressing content, time, method and place of delivery [22, 26].

Our study has many strengths. First, this was a prospective examination of a large population of low-risk nulliparous pregnant women. Second, there was data recording of maternal characteristics and medical history to identify risk factors associated with preeclampsia. Furthermore, we measured arterial blood pressure with a manual sphygmomanometer which was standardized in the protocol for noninvasive blood pressure monitoring [6]. On the other hand, it is known that there is concern about the clinical performance and safety of these instruments. Although BMI had been registered at enrolment, a weakness of our study was that participants were not segregated by weight. This can add a significant bias, despite the exclusion of women with comorbidities. Obesity was twofold higher among women with preeclampsia than among controls. Nevertheless, weight was not an exclusion factor. MAP is known to be dependent on weight [27]. However, a cuff of the appropriate size for each patient weight was selected for blood pressure measurement. Another important limitation of our study was the very few cases of early-onset preeclampsia, as our cohort was built with healthy participants. This can impact the results, weakening some analysis. This is why we consider future studies with larger cohorts to be important.

## Conclusion

In the last 20 years, improvement in diagnostic tools has led to the prevention and prediction of different conditions, including obstetric health issues. Therefore, considerable effort has been devoted to identifying and modifying individual risk factors during the first half of pregnancy [25, 26]. Close surveillance of the mother and offspring may help to decide the best time for delivery. Furthermore, owing to the complexity of preeclampsia and its multifactorial etiology, the predictive power seems to be correlated with the association of distinct factors in a prediction model, and is not related to a unique isolated risk factor or biomarker [28]. Therefore, future studies should be conducted to analyze mean arterial blood pressure in combination with other strategies, to obtain a predictive algorithm for preeclampsia, particularly in the challenging nulliparous group.

## Data Availability

The datasets generated and analysed during the current research are available from the corresponding author on reasonable request. The participating women did not give their consent to make their own data publicly available.

## Abbreviations

AUROC: Area under the receiver-operating characteristic curves
BP: Blood pressure
DBP: Diastolic blood pressure
EO-PE: Early-onset preeclampsia
LO-PE: Late-onset preeclampsia
MAP: Mean arterial blood pressure
PE: Preeclampsia
SBP: Systolic blood pressure
